# Descriptive Analysis of Good Clinical Practice Inspection Findings from the Saudi Food and Drug Authority

**DOI:** 10.1007/s43441-024-00731-5

**Published:** 2024-12-26

**Authors:** Omaima O. Arab, Mohammed Aldayan, Khalid Almowaizri, Ahmad Alghamdi, Jahad Alghamdi, Adel Alharf

**Affiliations:** Clinical Trials Department Benefit and Risk Assessment Executive Directorate Drug Sector - Saudi Food and Drug Authority, Riyadh, 13513– 7148 Saudi Arabia

**Keywords:** Clinical trial, Regulatory, Inspection, Investigation, Good clinical practice, Saudi food and drug authority, SFDA

## Abstract

**Introduction:**

The Saudi Food and Drug Authority (SFDA) conducts inspections in accordance with Good Clinical Practice (GCP) to safeguard clinical trial integrity and protect the rights, safety, and welfare of study participants. These inspections ensure that trials are conducted in compliance with GCP and applicable laws.

**Objectives:**

The study aims to provide a description of GCP inspection findings, analyze their impact on the clinical trial ecosystem, and provide recommendations to improve clinical trial conduction in Saudi Arabia.

**Methods:**

A review was conducted on inspection reports, with two senior independent inspectors examining, collecting, and categorizing the data. Descriptive statistics were used to summarize the categorical variable via frequency distributions.

**Results:**

A total of 131 GCP inspections were performed between 2017 and 2023, totaling 722 observations from 116 (88.5%) inspection visits. The remaining 15 (11.5%) inspection visits recorded no observations. The highest number of visits were conducted in contract research organizations (CRO) (*n* = 50; 38.2%) with 118 observations, followed by clinical investigator sites (*n* = 46; 35.1%) with 313 observations, then bioequivalence (BE) centers (*n* = 33; 25.2%) with 256 observations, and the last 2 (1.5%) visits were conducted in phase I clinical trial units with 35 observations.

**Conclusion:**

This study assesses GCP inspection reports and examines the types of deficiencies and their grades in each area. Observation categories and grades were found to vary by organization type, which indicates the need for specific action plans addressing each organization type separately. This report provided recommendations based on the most common findings to assist researchers and sponsors when conducting clinical trials in Saudi Arabia.

**Supplementary Information:**

The online version contains supplementary material available at 10.1007/s43441-024-00731-5.

## Introduction

Good Clinical Practice (GCP) is an international ethical and scientific quality standard for designing, conducting, recording, and reporting trials that involve the participation of human subjects [1]. GCP inspections play a vital role in ensuring compliance and providing public assurance that the rights, safety, and well-being of the individuals participating in clinical trials are safeguarded and that clinical trial data is credible [2, 3]. The Saudi Food and Drug Authority (SFDA) conducts GCP inspections to uphold the integrity of clinical trials, protect the rights, safety, and welfare of study participants, and ensure that trials are conducted in compliance with GCP and applicable laws and regulations [4, 5]. To facilitate clinical trials in Saudi Arabia, SFDA has developed, adopted, and published various regulatory documents and guidance, such as the Regulations and Requirements for Conducting Clinical Trials on Drugs [6].

GCP inspections utilize a data-focused approach, verifying individual subject-level data and the overall conduction of clinical trials at clinical investigator sites, bioequivalence (BE) centers, and contract research organizations (CRO). They are conducted on drug studies and facilities performing clinical trial activities by the drug sector. Reports and observations from inspection visits are archived for documentation purposes. The SFDA inspection team at the clinical trial department has gathered data from GCP inspections. Detailed analysis and study of this data was performed to share the Saudi GCP inspection experience and provide recommendations to improve the clinical trial ecosystem.

This report aims to describe GCP findings from sites inspected by the SFDA from 2017 to 2023. It discusses the results and their implications in comparison to international counterparts and reflects on the results with recommendations for investigators to improve clinical trial conduction in KSA.

### SFDA Inspection Program

The Clinical Trial Department at SFDA was established in 2009 [5]. It is this department’s responsibility to provide informed decisions on registered clinical trials and approve conductions in Saudi Arabia [5]. In addition, GCP inspections have been introduced to ensure regulatory compliance and trial integrity [5]. In addition, one of the requirements of accepting bioequivalence studies is that the BE center is accredited and inspected by SFDA. Planned inspections are carried out to surveil GCP compliance, whether by routine or registration visits, in the absence of specific issues. Non-routine inspections are triggered by issues arising during the course of the trial. Both planned and triggered inspections are conducted in a systematic approach where a team of three inspectors, typically, are assigned to visit sites. There are seven main GCP observation categories: principles, regulatory requirement, investigator (research team), sponsor, clinical trial protocol, investigator brochure, and essential documents, as defined and listed in Table 1.

**Table 1:** GCP inspection findings by deficiency areas

GCP findings	Definitions
The principles	ICH GCP principles include 14 points reflecting the protection of human rights, scientific justification of trial, determination of risks and benefits, guided by prospect of benefit, approved by IRB/EC, complied with protocol, provided participants informed consent, under qualified and licensed medical team, trained and educated research team, appropriate documentation, privacy and confidentiality of information, investigational product complies with GMP, and system to ensure quality of trial features.
Regulatory requirement	Applicable Regulatory Requirement(s) Any law(s) and regulation(s) addressing the conduct of clinical trials of investigational products.
Investigator (Research team)	A person responsible for conducting the clinical trial at a trial site. If a trial is conducted by a team of individuals at a trial site, the investigator is the responsible leader of the team and may be called the principal investigator.
Sponsor	**1.53 Sponsor** An individual, company, institution, or organization that takes responsibility for the initiation, management, and/or financing of a clinical trial. **1.54 Sponsor-Investigator** An individual who both initiates and conducts, alone or with others, a clinical trial and under whose immediate direction the investigational product is administered to, dispensed to, or used by a subject. The term does not include any person other than an individual (e.g., it does not include a corporation or an agency). The obligations of a sponsor-investigator include both those of a sponsor and those of an investigator.
Clinical trial protocol	A written description of a trial/study of any therapeutic, prophylactic, or diagnostic agent conducted in human subjects, in which the clinical and statistical description, presentations, and analyses are fully integrated into a single report
Investigators Brochure	A compilation of the clinical and nonclinical data on the investigational product(s) that is relevant to the study of the investigational product(s) in human subjects
Essential Documents	Documents which individually and collectively permit evaluation of the conduct of a study and the quality of the data produced

Data from visit are collected, and findings are documented and classified according to established criteria. Then, a report is generated to address all findings and shared with the investigators within 21 working days. The investigators are to respond within 60 working days if a Corrective and Preventative Action (CAPA) plan(s) is required [7].

### Good Clinical Practice Inspection

The clinical trial department’s inspection-related responsibilities include but are not limited to: organizing and coordinating the inspection process, conducting the inspection, preparing the inspection report, reviewing CAPA plan(s), and finalizing the inspection. Investigators have to ensure that clinical trials are conducted in compliance with SFDA regulations and the relevant GCP guideline(s), maintain readiness for inspection (as inspections may be unannounced), provide inspectors with any information or documentation they need to prepare or conduct the inspection, ensure staff involved in the clinical trial are available during the inspection for interviews or to clarify issues, and prepare and implement appropriate and timely CAPA plans to address the inspection’s findings and prioritize any critical or major deficiencies.

The SFDA’s GCP inspection program has been designed to harmonize with current international approaches. Trials to be inspected are selected based on risk-based internal criteria focusing on the trial phase, type of investigational drug, category of trial participants, and other safety measures. The selection is based on a scoring system according to the collective highest count number from the Guiding Risk Assessment Approach [7]. Therefore, some sites may be inspected more than once for different trials and/or studies. Examples of high-score trials include trials conducted during pandemics, trials conducted on vulnerable subjects, phase I trials, vaccine trials, and trials for advanced therapeutic medicinal products (ATMPs).

### Methods

The current study has an Institutional Review Board (IRB) exempt from the local IRB committee at SFDA (SFDA approval number 2023_03). The data sources used for this project are the SFDA’s inspection records, GCP inspection report forms, and individual inspection reports. Two senior independent inspectors reviewed inspection data and individual reports to gather and categorize the information. An additional senior clinical trial expert verified all the data. The department’s internal database is the resource of documented and approved data in this study. The formal launch of the SFDA’s GCP inspections was in 2015; however, only data from 2017 to 2023 was included. The main reasons for the exclusion of data prior to 2017 are premature systems, unstructured inspection processes, inadequate inspector experience, a low number of sites, and the introduction of quality improvement processes.

The GCP inspection findings follow the SFDA GCP guideline adopted from the ICH Guideline for Good Clinical Practice E6(R2) [3]. Identified deficiencies are classified based on significance (critical, major, other, and comments) and as per PIC/S Guidance on Classification of GMP Deficiencies [8].

### Analysis

Categorical variables are summarized using descriptive statistics by frequency distributions (i.e., number and percentage of subjects within a given category in the analysis data set). Each inspection visit is considered an individual GCP inspection regardless of the visited site.

## Results

**Fig. 1:**
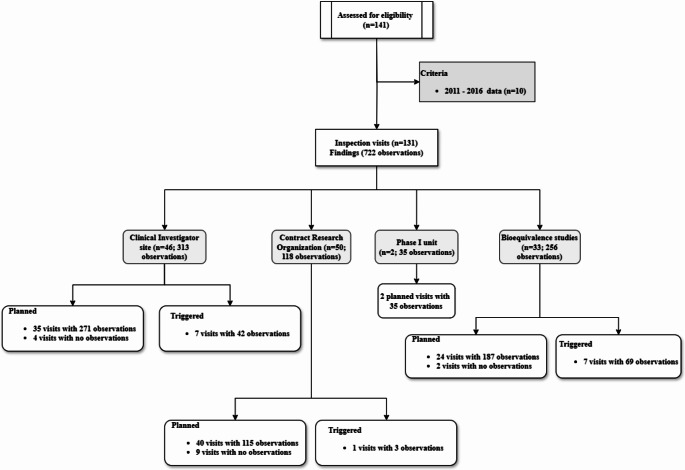
Flowchart of data and results

**Fig. 2:**
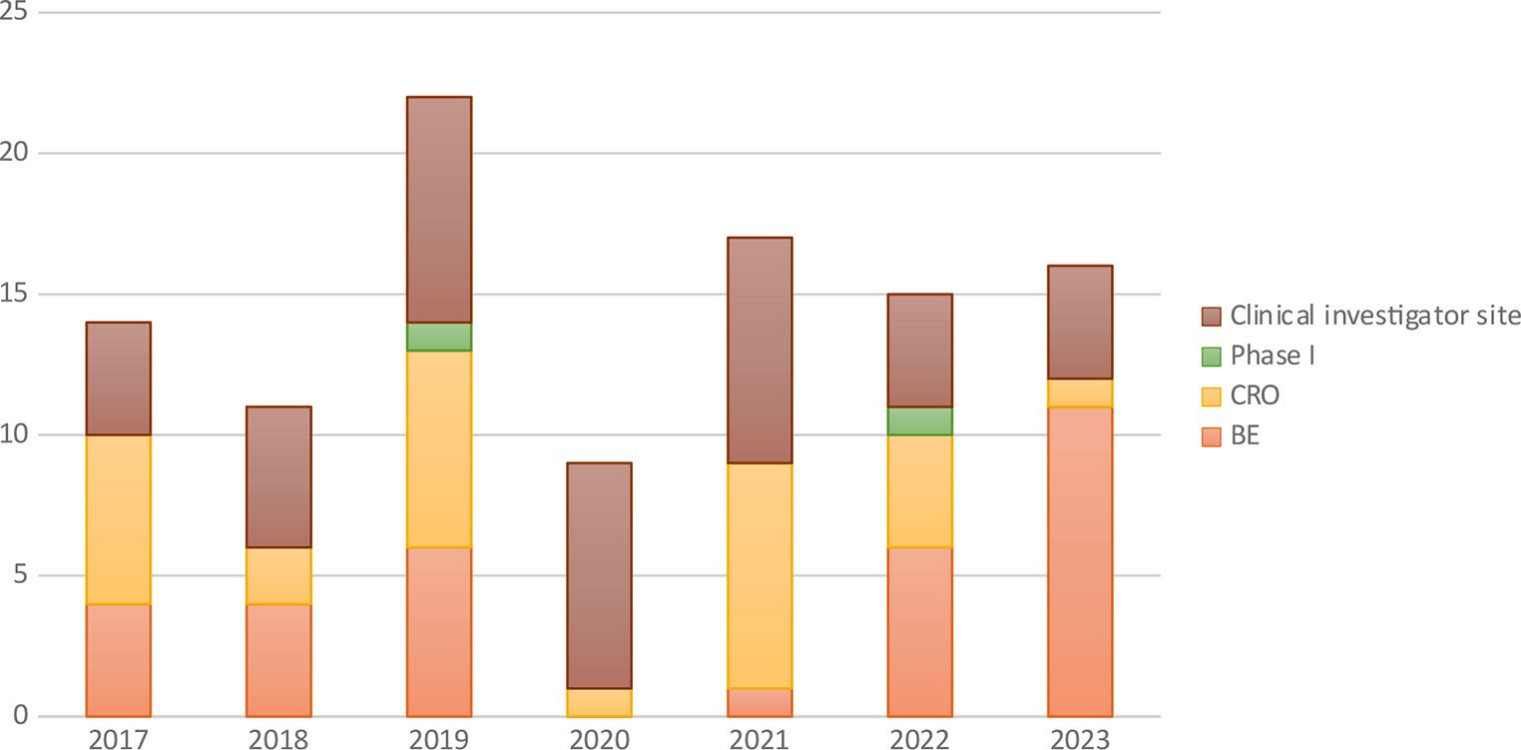
Number of GCP inspection visit per year (2017–2023)

A total of 141 inspection visits have been performed since launching the program. Between 2017 and 2023, a total of 131 GCP inspections were performed (Fig. 1). Of them, our inspectors reported a total of 722 observations from 116 (88.5%) inspection visits, and the remaining 15 (11.5%) inspection visits reported no observations. Classifying the inspection visits by organization type shows that the highest number of visits were conducted in CROs (*n* = 50; 38.2%), followed by clinical investigator sites (*n* = 46; 35.1%), then BE centers (*n* = 33; 25.2%) and the last 2 (1.5%) visits were conducted in phase I clinical trial units (Fig. 2).

**Fig. 3:**
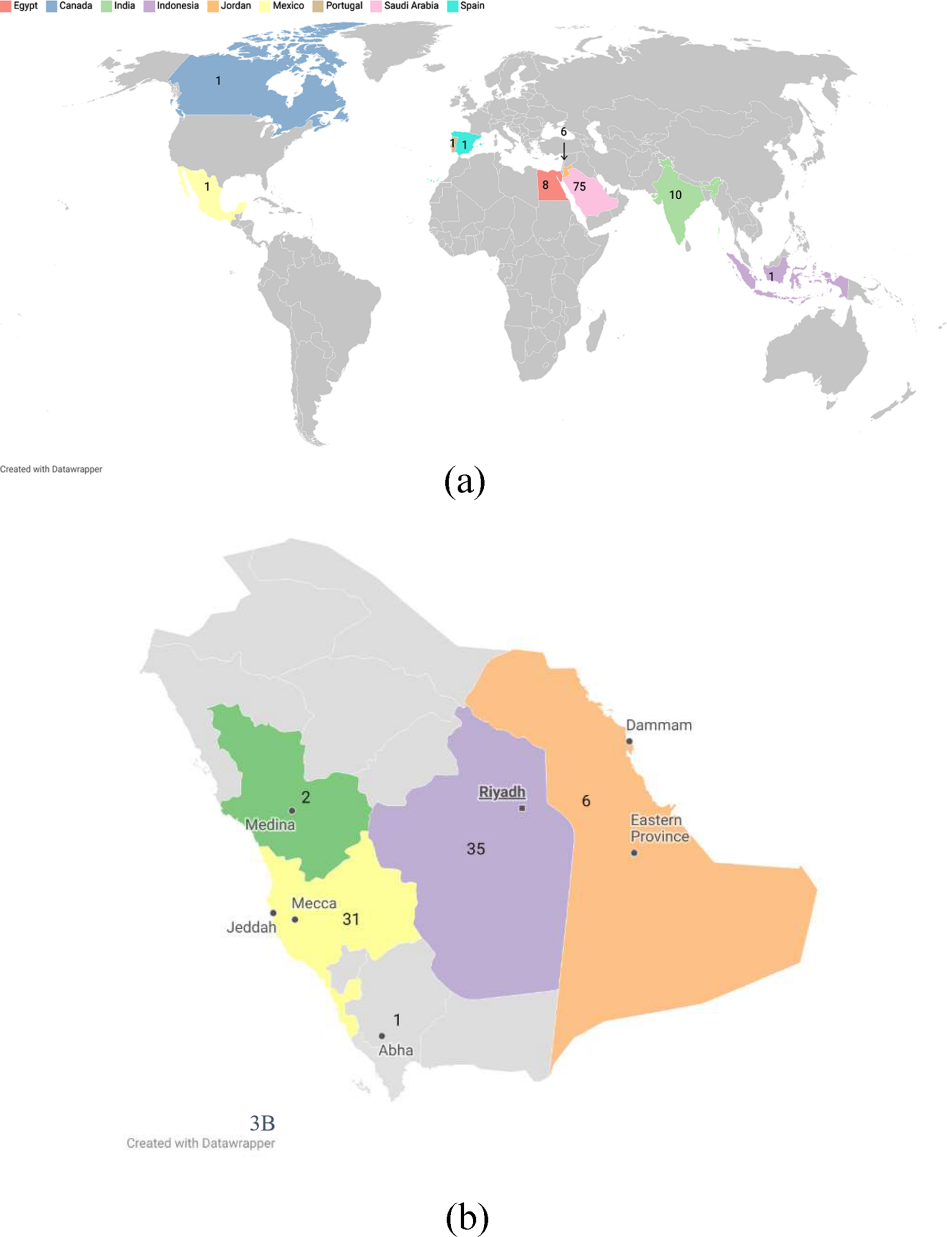
Geographical distribution of inspection visits (3 A: international, 3B: national)

Inspections were conducted nationally and internationally. Almost all the BE center inspections were conducted internationally in the following countries: India, Jordan, Egypt, Portugal, Spain, Canada, and Mexico. Other inspections were conducted nationally in several cities, including Riyadh, Jeddah, Makkah, Al-Khobar, Dammam, Al-Ahsa, Abha, and Madinah. (Fig. 3).

**Fig. 4:**
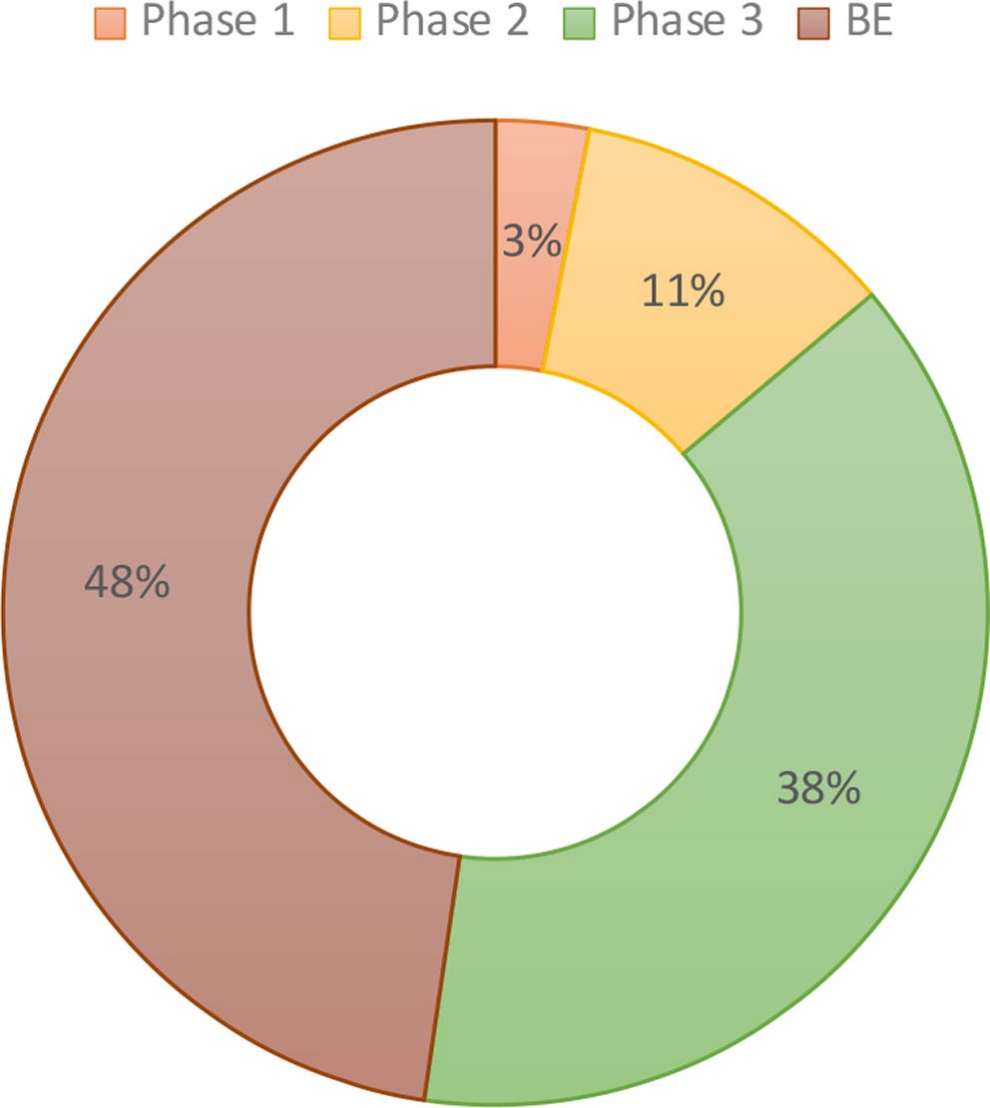
Percentage of clinical trial phases and BE studies at inspection visits

Inspections were mostly conducted on BE studies (*n* = 33; 48%), followed by phase III (*n* = 25; 38%), phase II (*n* = 7; 11%), and phase I (*n* = 2; 3%) clinical trials (Fig. 4**)**. The most frequent therapeutic area to be inspected was hematology-oncology, followed by general hematology, then cardiology, and finally emergency medicine.

In terms of the number of observations, clinical investigator sites accounted for the highest observation count of 313 (43.5%) from 46 inspection visits, followed by BE centers totaling 256 observations (35.5%) from 33 inspection visits. CROs were associated with 118 observations (16%) from 50 inspection visits, while phase I clinical trial units had the fewest observations, with 35 (5%) from 2 inspection visits.

### Observations at Clinical Trial Investigator Sites

**Fig. 5:**
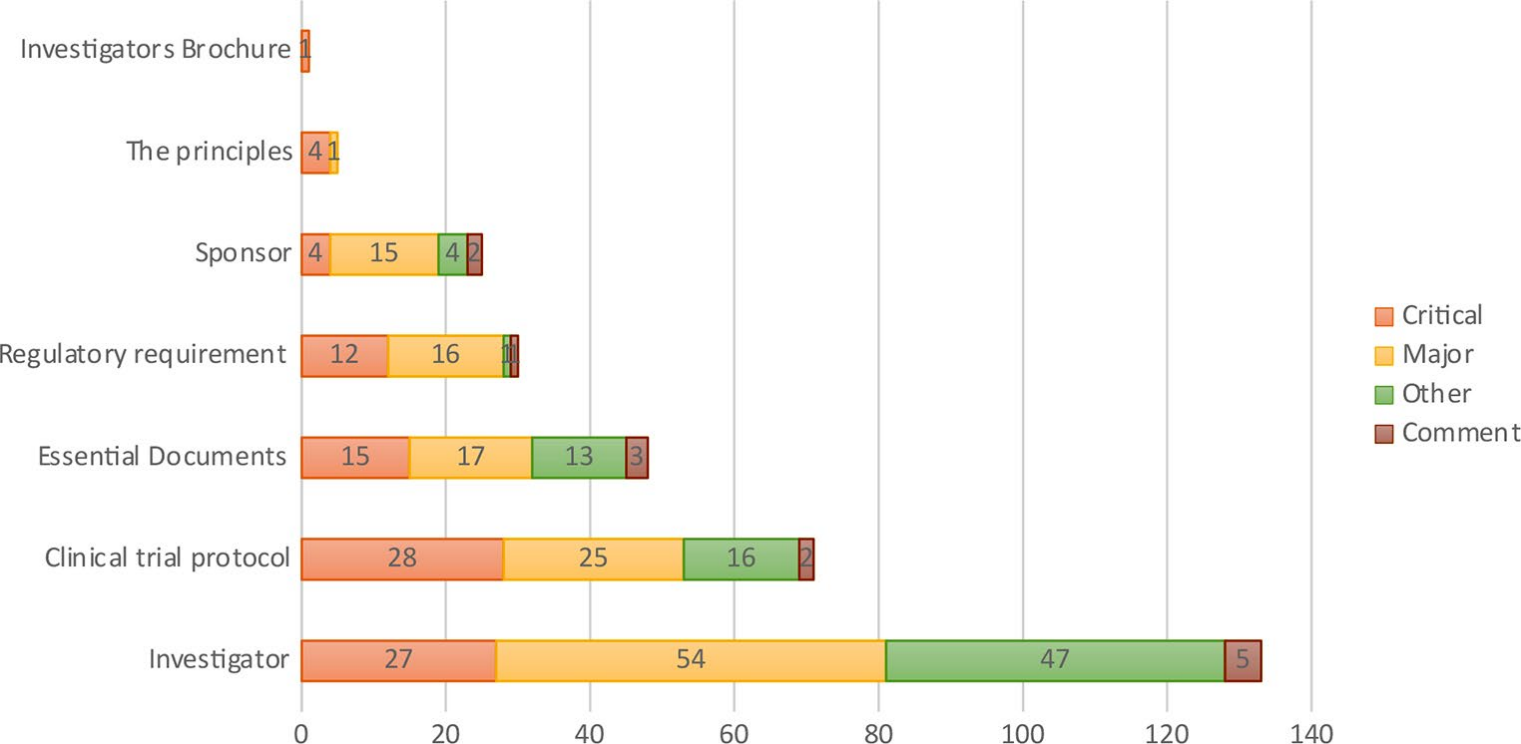
GCP observations and grades at clinical trial investigator sites

A total of 46 inspection visits were conducted at clinical investigator sites, and 313 observations were reported from all of them. Four inspection visits reported no observations. The top three observation categories per GCP guidelines were Investigator (*n* = 133; 42.5%), followed by Clinical Trial Protocol (*n* = 76; 24.3%) and Essential Documents (*n* = 48; 15.3%). Classifying the findings by grade revealed the following distribution: critical (*n* = 91; 29%), major (*n* = 128; 40.9%), other (*n* = 81; 25.9%), and comments (*n* = 31; 4.1%) (Fig. 5).

### Observations at CROs

**Fig. 6:**
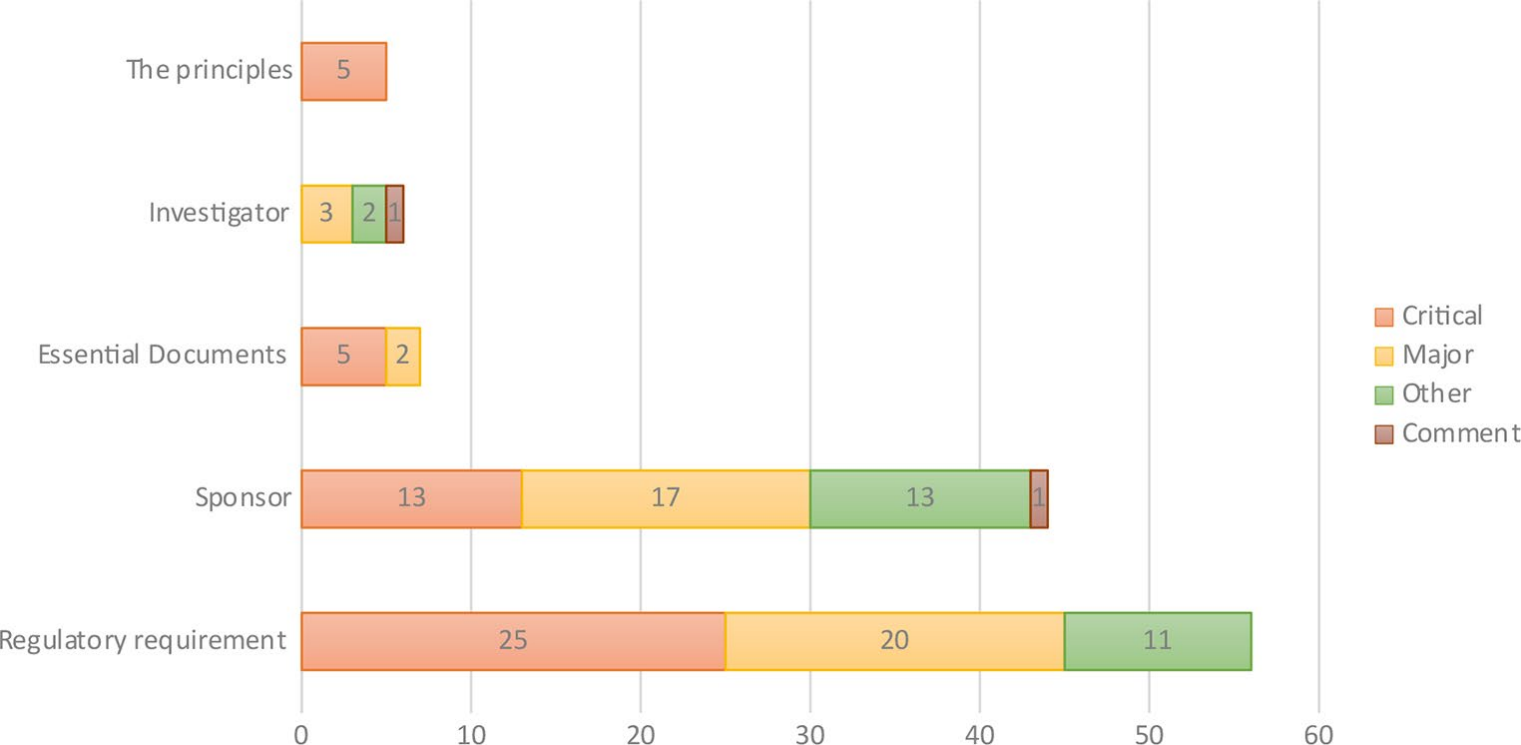
GCP observations and grades at CROs

A total of 50 inspection visits were conducted at CRO sites, with 118 observations reported from all of them. No observations were reported from nine inspection visits. The top observation categories per GCP guidelines were Regulatory Requirement (*n* = 56; 47.5%), followed by Sponsor-related observations (*n* = 44; 37%). Classifying the findings by grade reveals the following distribution: critical (*n* = 48; 40.5%), major (*n* = 42; 35.6%), other (*n* = 26; 22%), and comments (*n* = 2; 1.7%) (Fig. 6).

### Observations at BE centers

**Fig. 7:**
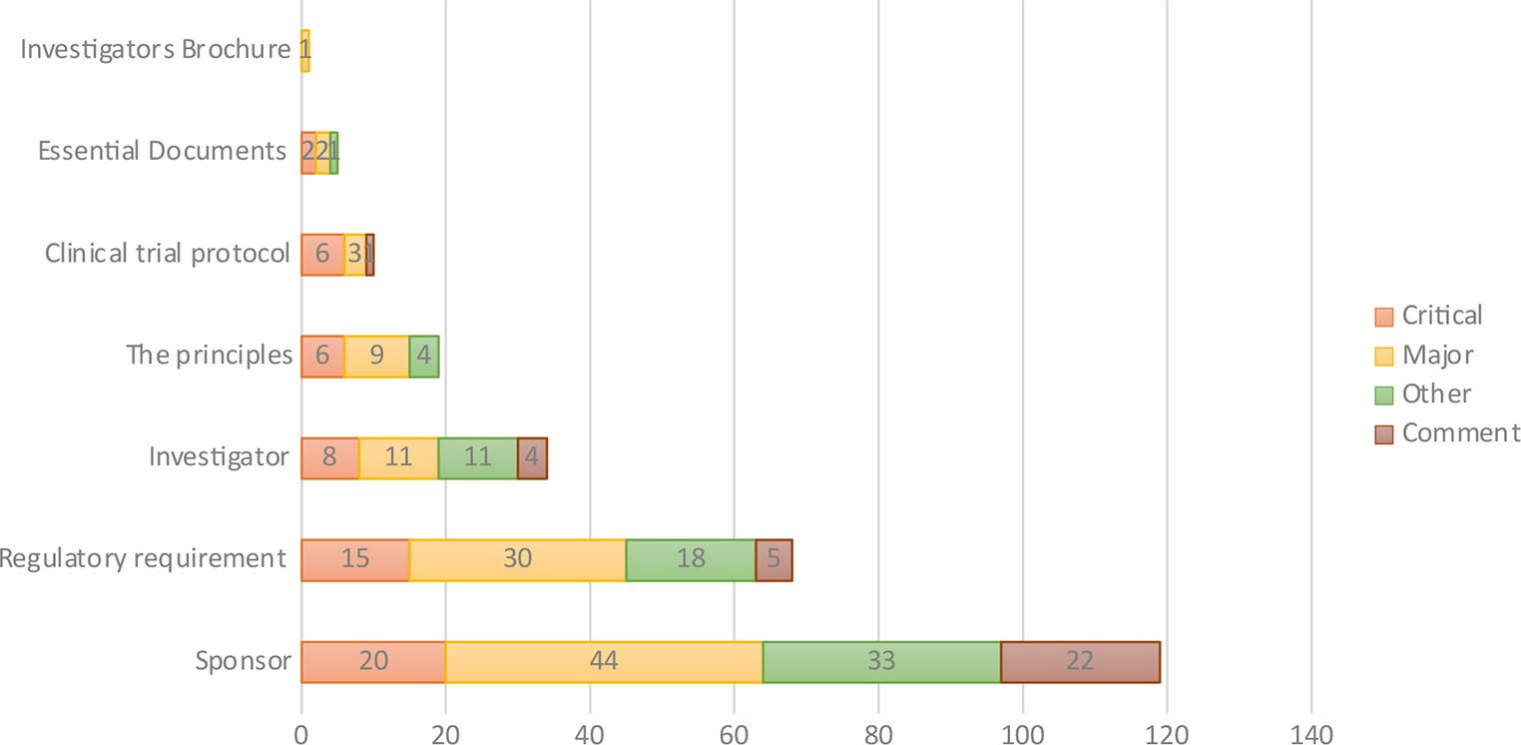
GCP observations and grades at BE centers

A total of 33 inspection visits were conducted at BE centers, with 256 observations reported from all of them. No observations were reported from two inspection visits. The top observation categories per GCP guidelines were related to Sponsor (*n* = 119; 46.5%), followed by Regulatory-requirement (*n* = 68; 26.5%). Classifying the findings by grade reveals the following distribution: critical (*n* = 57; 22.3%), major (*n* = 100; 39%), other (*n* = 67; 26%), comments (*n* = 32; 12.5%) (Fig. 7).

### Observations at Phase I Units

**Fig. 8:**
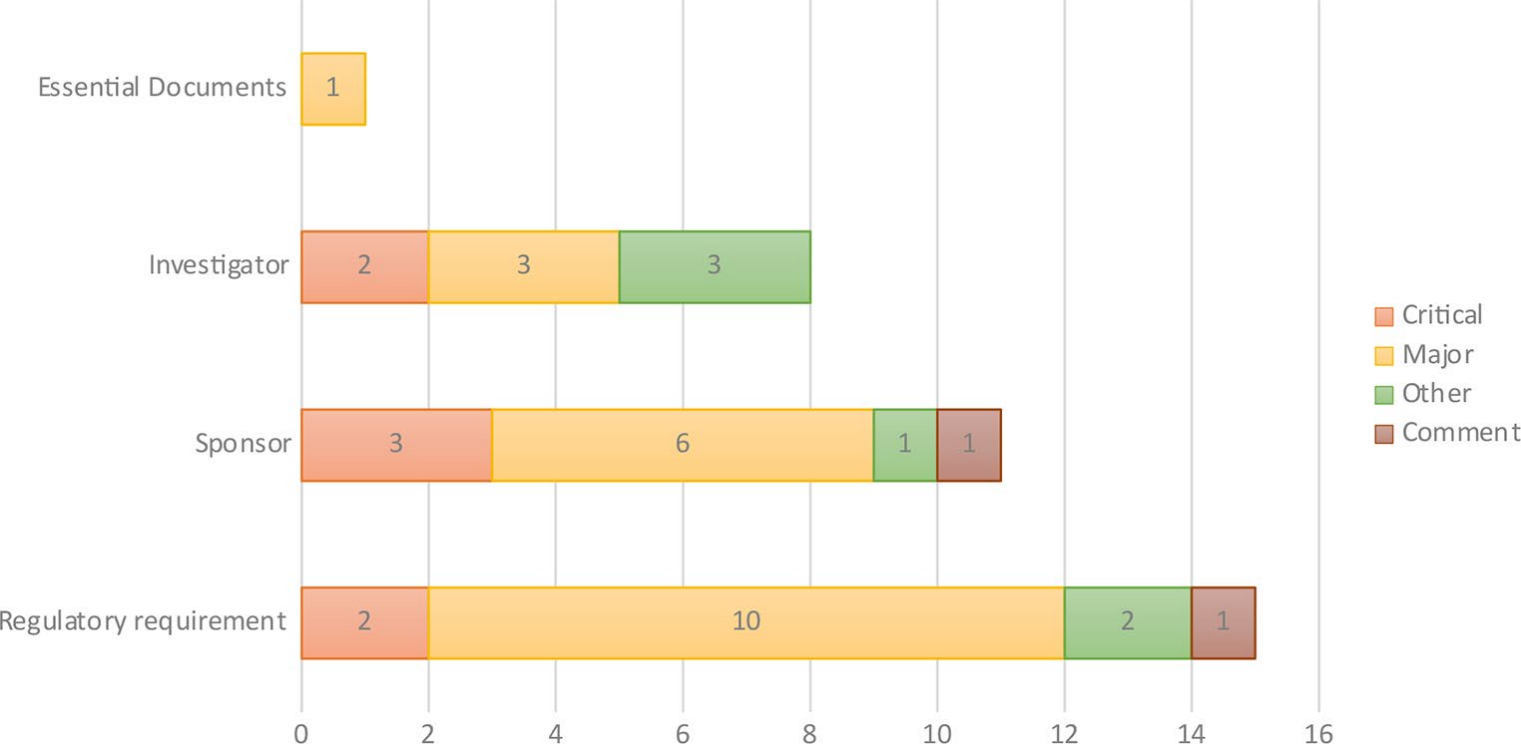
GCP findings and grades at phase 1 units

Two phase I trial units were inspected, accounting for 35 observations, primarily in the categories of Regulatory requirement (*n* = 15; 42.9%) and Sponsor (*n* = 11; 31.5%). Classifying the findings by grade revealed the following distribution: critical (*n* = 7; 20%), major (*n* = 20; 57%), other (*n* = 6; 17%), and comments (*n* = 2; 5.7%) (Fig. 8).

## Discussion

The purpose of this study is to report the findings of GCP visits conducted by SFDA inspectors since the official implementation of the inspection program in 2017. The GCP inspection program implemented by SFDA has been developed to align with contemporary international guidelines. The selection of trials for inspection is determined by internal risk-based assessment criteria, which consider several factors such as trial phase, type of investigational drug, trial cohort, and other safety measures. Since the implementation of the GCP inspection program in 2017, the number of GCP visits has escalated, reaching a peak of 22 visits in 2019, followed by 17 visits in 2021. This is in accordance with the observed growth in the number of registered clinical trials in Saudi Arabia in recent years. The increase is evident in both primary-investigator-initiated trials and sponsored clinical trials. The value of this report is to identify areas of deficiency, allowing relevant stakeholders to evaluate and devise an effective action plan.

Inspection visits show that CROs had the most visits, followed by clinical investigator sites and BE centers (50, 46, and 33 visits, respectively). Phase I trial units had the fewest visits (2 visits). Highest number of inspections seems to be driven by CROs and clinical investigator site inspections which could be due to approximate location and type of application. In contrast, the vast majority of BE inspections were conducted globally. While phase I units are still few, the SFDA has visited two sites for guidance and accreditation, primarily while visiting the unit and meeting research teams. Regarding therapeutic areas, oncology clinical trials were the most inspected trials, which is expected due to the risk-based approach for prioritizing inspection, this also could highlight an observation that cancer research seems to lead clinical trials in Saudi. Complex and advanced trials including cell and gene therapies were inspected as noted.

The findings show diverse observations related to each area inspected. For example, Sponsor and Regulatory Requirement observations were highest at CROs and BE centers which can be attributed to the fact that most of these inspection visits were for registration purposes. Similarly, Regulatory Requirement observations were high at phase I trial units due to the first-visit experience at the units and initial setup improvement requirements. However, this observation category is among the lowest at clinical investigator sites, which may reflect better compliance with GCP guidance. In addition, most of these sites harbor Clinical Trials Units (CTUs) that are expected to facilitate conduction of clinical trials within these institutions, and ensure the compliance with GCP.

In terms of observation grades, attaining a lower count of “critical” grades compared to “major” and “others” indicates that the identified findings might not adversely affect the rights, safety, or well-being of the subjects or the quality and integrity of data.

For critical observations, we have noticed a great decline trend of the number of critical observations over the years, were despite the fact that the number of inspection visits remained the same, the number of critical observations at clinical investigator sites, for examples, it reduced from around 22 critical observations during 2021 to only four critical observations during 2024. This indicate that the current local GCP training activities are improving the GCP compliance at the clinical investigator sites.

This report utilizes the same deficiency categories as those reported by other regulatory agencies, such as the United States Food and Drug Administration (FDA) and the European Medicines Agency (EMA) [2]. This enables comparability of the observed findings with those reported by other regulatory authorities, fostering a broader understanding of regulatory practices and harmonization. The result from Sellers et al. shows that the FDA found that Protocol Compliance for clinical investigator inspections and Trial Management issues for sponsor/contract research organization inspections were the most common GCP finding. While EMA report found deficiencies related to Documentation as the most common findings for both clinical investigator and sponsor/contract research organization inspections [2]. The result from the current study shows different observations in both areas. For Clinical Investigator Site, the most common findings are related to the investigator then to the Clinical Trial Protocol. While for CRO, the most common findings are Regulatory Requirement then Sponsor related findings. Therefore, this report suggests that focused GCP training courses should be initiated to educate research team and facilities about the GCP practices. Additionally, higher number of GCP inspections at clinical investigator sites should be conducted to observe the compliance with GCP standards.

Achieving robust GCP compliance requires a multi-pronged approach. Regular custom training for researchers on ethical considerations and GCP principles is essential. Furthermore, fostering a culture of transparency and accountability within research teams, along with improved communication between researchers, ethics committees, and regulatory bodies, will promote GCP adherence. Finally, implementing efficient data management systems and rigorous monitoring procedures will ensure data integrity and participant well-being, strengthening the research’s foundation and scientific value.

The results of the current study serve as a baseline for GCP inspections in Saudi Arabia, with the intention of establishing a strong foundation for these inspections and enhancing the overall clinical trial environment in Saudi Arabia. By understanding the specific challenges faced in each area during inspections, it becomes possible to address the observations effectively through the implementation of appropriate corrective and preventive actions. Further research is anticipated to compare observations and gradings of inspection areas and sites. The findings of this activity will be communicated in a targeted approach with each stakeholder or institutions in order to develop a working plan that address the required area of improvements per institution.

Based on a thorough evaluation of the results, SFDA has proposed a comprehensive set of recommendations to address the identified deficiencies and promote continuous improvement in the clinical trial landscape. In addition, as noted that some triggered visits resulted in critical observations mainly due to starting clinical trials prior to obtaining SFDA’s approval. The proposed recommendations include: raising awareness among researchers and clinical trial teams about the importance of GCP compliance, engaging patients in the clinical trial process to create a greater understanding and appreciation of clinical research, expanding clinical research training courses for research team members, implementing quality improvement projects to enhance study site infrastructure, and lastly, engaging and connecting stakeholders, including researchers, policymakers, regulatory authorities, patient groups, and industry representatives, from various sectors to work together in improving the clinical trial ecosystem in Saudi Arabia.

There were some limitations to our study that should be acknowledged. Firstly, it is important to note that this study relied on a retrospective analysis of GCP inspection data, making it subject to inherent limitations associated with this type of analysis. Additionally, this is the first report within SFDA and the region on GCP findings, which may introduce unique contextual factors that should be considered. Furthermore, the collection and allocation of data posed certain difficulties, which may have influenced the accuracy and completeness of the dataset. It is important to consider that various factors could have contributed to the observed differences in inspection findings. For instance, the inspector’s subjectivity, as well as their individual training, background, and expertise in the field of GCP inspections, could have influenced the outcomes. Despite these limitations, the study provides valuable insights into GCP inspections and lays the foundation for future research and improvement in this area. It is crucial to recognize these limitations and consider them when interpreting the results and drawing conclusions from the study.

## Conclusion

This study examines GCP inspection reports and provides an analysis of the types of deficiencies and their grades across four clinical trial sites: clinical trial investigator sites, CROs, BE centers, and phase I clinical trial units. Observation categories and grades varied across each organization type, indicating the need for specific action plans addressing each type separately.

Based on the most commonly identified findings, our study offers recommendations aimed at assisting researchers and sponsors in conducting successful clinical trials in Saudi Arabia. To advance this objective, we propose the implementation of joint GCP workshops, active engagement with global regulatory bodies during professional society conferences, participation in scientific exchange programs, and staying updated on the ICH-E6(R3) GCP guidelines. By adopting these strategies, we can foster a culture of continuous improvement and ensure that clinical trials in Saudi Arabia adhere to the highest standards of quality, ethics, and patient safety. The ultimate goal is to enhance the country’s clinical research landscape and contribute to the advancement of medical knowledge.

## Electronic Supplementary Material

Below is the link to the electronic supplementary material.

Supplementary Material 1

## Data Availability

No datasets were generated or analysed during the current study.
