# Supplementary material for: Descriptive Analysis of Good Clinical Practice Inspection Findings from the Saudi Food and Drug Authority
Source: Ther Innov Regul Sci. 2024 Dec 26;59(2):295–303. doi: 10.1007/s43441-024-00731-5 (PMC11880033; doi:10.1007/s43441-024-00731-5)
Supplement: Supplementary file 1 — Supplementary Material 1 [file 43441_2024_731_MOESM1_ESM.docx]

Descriptive Analysis of Good Clinical Practice Inspection Findings from the Saudi Food and Drug Authority

Supplementary

Omaima O. Arab

2024

**Contents**

[Table 1: GCP inspection findings by deficiency areas 3](#_Toc160618831)

[Table 2: Grading deficiencies and definitions 5](#_Toc160618832)

[Table 3: GCP Distribution of findings at clinical investigator site inspections 10](#_Toc160618833)

[Table 4: GCP distribution of findings at CRO inspections 11](#_Toc160618834)

[Table 5: GCP distribution of findings at BE inspections 12](#_Toc160618835)

[Table 6: GCP distribution of findings at Phase I units inspections 13](#_Toc160618836)

[Figure 1: Good Clinical Practice visits and observation (findings) 6](#_Toc160618837)

[Figure 2: Number of planned inspections visit per year (2017-2023) 7](#_Toc160618838)

[Figure 3: Geographical distribution of inspection visits (3A: global, 3B: national) 8](#_Toc160618839)

[Figure 4: Percentage of phases/BE at inspection visits 9](#_Toc160618840)

[Figure 5: Therapeutic areas and phases of inspected facilities 10](#_Toc160618841)

[Figure 6: Clinical investigator site GCP observations and grades 11](#_Toc160618842)

[Figure 7: CRO GCP observations and grades 12](#_Toc160618843)

[Figure 8: BE GCP observations and grades 13](#_Toc160618844)

[Figure 9: Phase I Units GCP observations and grades 14](#_Toc160618845)

**Table 1: GCP inspection findings by deficiency areas ^[[1]](#footnote-1)^**

| **GCP findings** | **Definitions** |
| --- | --- |
| The principles | ICH GCP principles include 14 points reflecting the protection of human rights, scientific justification of trial, determination of risks and benefits, guided by prospect of benefit, approved by IRB/EC, complied with protocol, provided participants informed consent, under qualified and licensed medical team, trained and educated research team, appropriate documentation, privacy and confidentiality of information, investigational product complies with GMP, and system to ensure quality of trial features. |
| Regulatory requirement | Applicable Regulatory Requirement(s)  Any law(s) and regulation(s) addressing the conduct of clinical trials of investigational products. |
| Investigator (Research team) | A person responsible for conducting the clinical trial at a trial site. If a trial is conducted by a team of individuals at a trial site, the investigator is the responsible leader of the team and may be called the principal investigator. |
| Sponsor | **1.53 Sponsor**  An individual, company, institution, or organization that takes responsibility for the initiation, management, and/or financing of a clinical trial.  **1.54 Sponsor-Investigator**  An individual who both initiates and conducts, alone or with others, a clinical trial and under whose immediate direction the investigational product is administered to, dispensed to, or used by a subject. The term does not include any person other than an individual (e.g., it does not include a corporation or an agency). The obligations of a sponsor-investigator include both those of a sponsor and those of an investigator. |
| Clinical trial protocol | A written description of a trial/study of any therapeutic, prophylactic, or diagnostic agent conducted in human subjects, in which the clinical and statistical description, presentations, and analyses are fully integrated into a single report |
| Investigators Brochure | A compilation of the clinical and nonclinical data on the investigational product(s) that is relevant to the study of the investigational product(s) in human subjects |
| Essential Documents | Documents which individually and collectively permit evaluation of the conduct of a study and the quality of the data produced |

- Principle 1: Research that involves humans should be scientifically sound and conducted in line with basic ethical principles originating from the Declaration of Helsinki. Three principles of equal importance associated with basic ethics, particularly recognized for persons, beneficence, and justice, permeate all other GCP principles.
- Principle 2: Research that involves humans should be described clearly in a written detailed protocol, and scientifically justified.
- Principle 3: Before the research that involves humans is begun, possible risks, obstacles, discomforts, and expected benefit(s) for the individual research subject and society should be determined.
- Principle 4: Research that involves humans should be started on the condition that the expected benefit(s) for the individual research subject and society clearly outweigh the risks.
- Principle 5: Research that involves humans should get approval/favorable opinion from the independent ethics committee/institutional review board (IEC/IRB) prior to initiation.
- Principle 6: Research that involves humans should be performed in compliance with the approved protocol.
- Principle 7: Freely given informed consent should be collected from each individual research subject prior to research participation in accordance with national culture(s) and requirements.
- Principle 8: Research that involves humans should be maintained only if the benefit-risk profile of the involved humans remains favorable.
- Principle 9: Qualified and duly licensed medical personnel should be responsible for the medical care of individual research subjects, and for any medical decision(s) made on their behalf.
- Principle 10: All individuals that are involved in a trial should be qualified by education, training, and experience to perform their respective task(s) and currently licensed to do so, where required.
- Principle 11: All clinical trial information should be documented, handled, and stored in a way that allows accurate reporting, interpretation, and verification.
- Principle 12: The confidentiality of information that could identify subjects should be secured, respecting the privacy and confidentiality rules in accordance with the applicable regulatory requirement(s).
- Principle 13: Investigational products should be manufactured, handled, and stored according to the applicable Good Manufacturing Practice (GMP) and should be used in accordance with the approved protocol.
- Principle 14: Systems with procedures that ensure the quality of every feature of the trial should be implemented.

**Table 2: Grading deficiencies and definitions^[[2]](#footnote-2)^**

| **Grading deficiencies** | **Definition** |
| --- | --- |
| Critical Deficiency | A deficiency which has produced, or leads to a significant risk of producing either a product which is harmful to the human or veterinary patient or a product which could result in a harmful residue in a food producing animal.  A “Critical” deficiency also occurs when it is observed that the manufacturer has engaged in fraud, misrepresentation or falsification of products or data.  A “Critical” deficiency may consist of several related deficiencies, none of which on its own may be “Critical”, but which may together represent *a” Critical*” deficiency, or systems’ failure where a risk of harm was identified and should be explained and reported as such. |
| Major Deficiency | A deficiency that is not a “Critical” deficiency, but which:   - has produced or may produce a product which does not comply with its Marketing Authorisation, Clinical Trial Authorisation, product specification; pharmacopoeia requirements or dossier; - does not ensure effective implementation of the required GMP control measures; - indicates a major deviation from the terms of the manufacturing authorisation; - indicates a failure to carry out satisfactory procedures for release of batches or (within PIC/S) failure of the authorised person to fulfil his/her duties; - consists of several “Other” related deficiencies, none of which on its own may be “Major”, but which may together represent a “Major” deficiency or systems failure and should be explained and reported as such. |
| Other Deficiency | A deficiency that is not classified as either “Critical” or “Major”, but indicates a departure from Good Manufacturing Practice (GMP).  A deficiency may be judged as “Other” because there is insufficient information to classify it as “Critical” or “Major”. |
| Comment | One-off minor discrepancies are usually not formally considered deficiencies, but are brought to the attention of the manufacturer as comments. |

**Figure 1: Good Clinical Practice visits and observation (findings)**


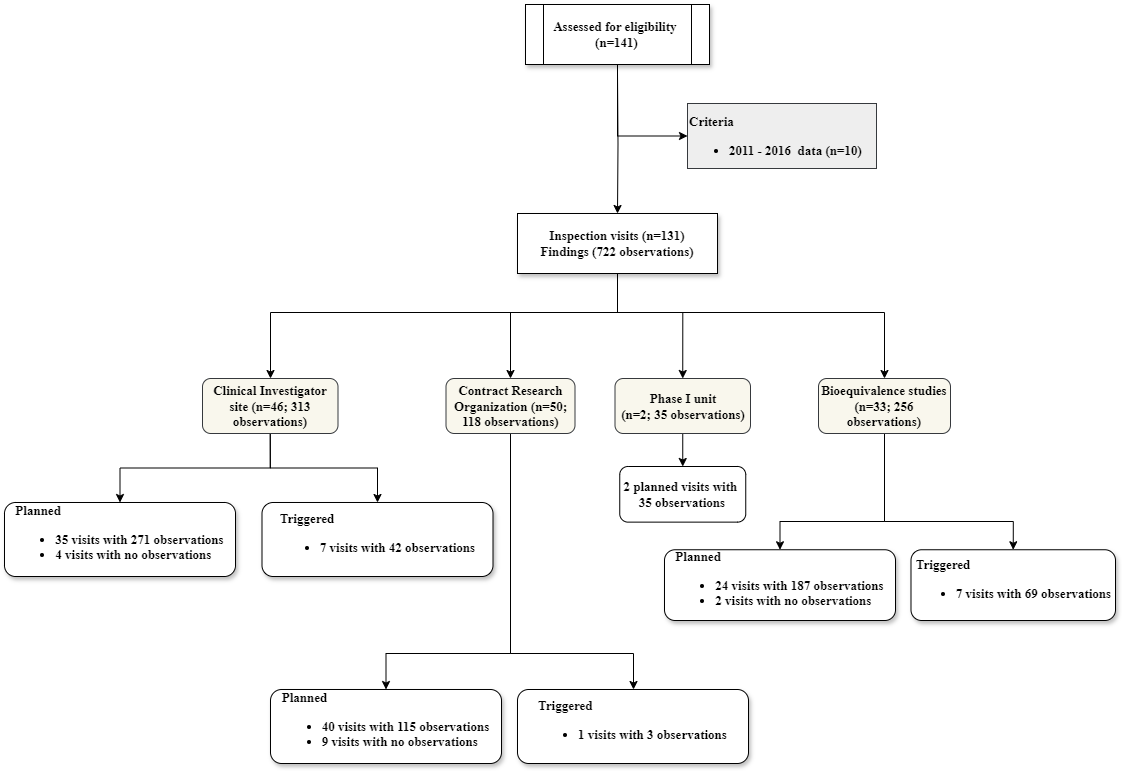


**Figure 2: Number of inspection visits per year (2017-2023)**

**Figure 3: Geographical distribution of inspection visits (3A: internatinal, 3B: national)**

3A

3B


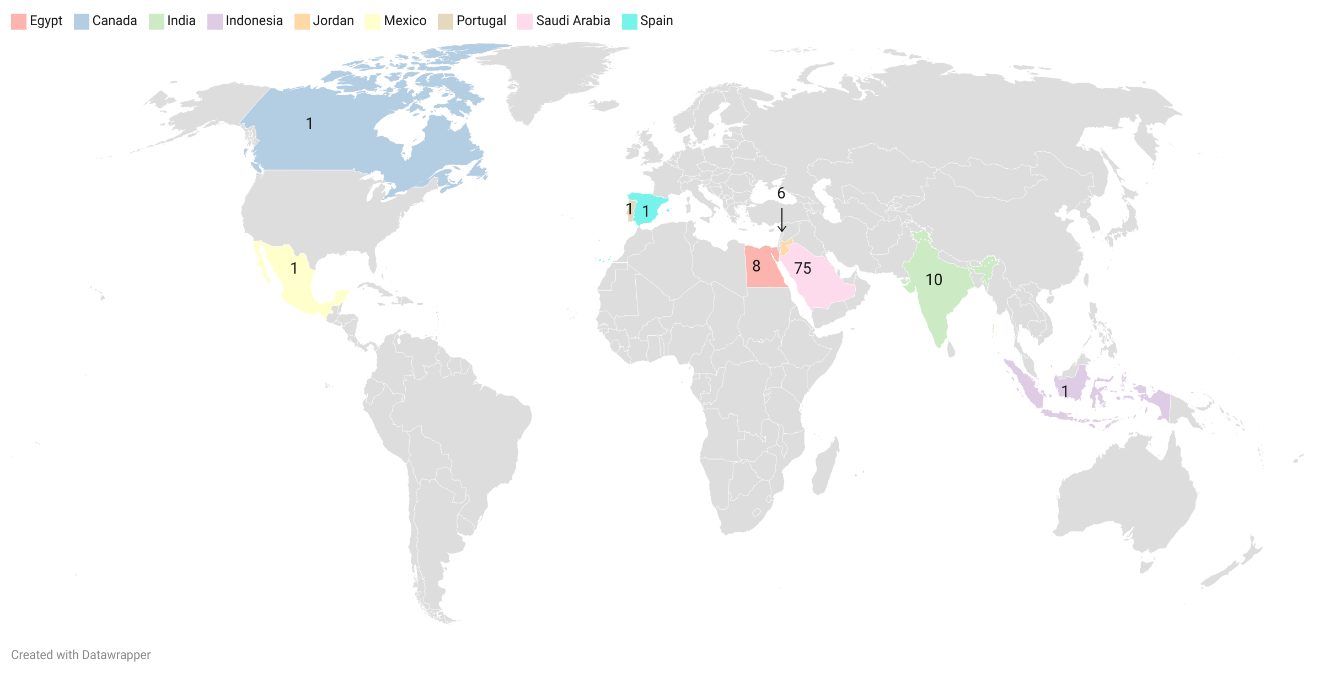


3A


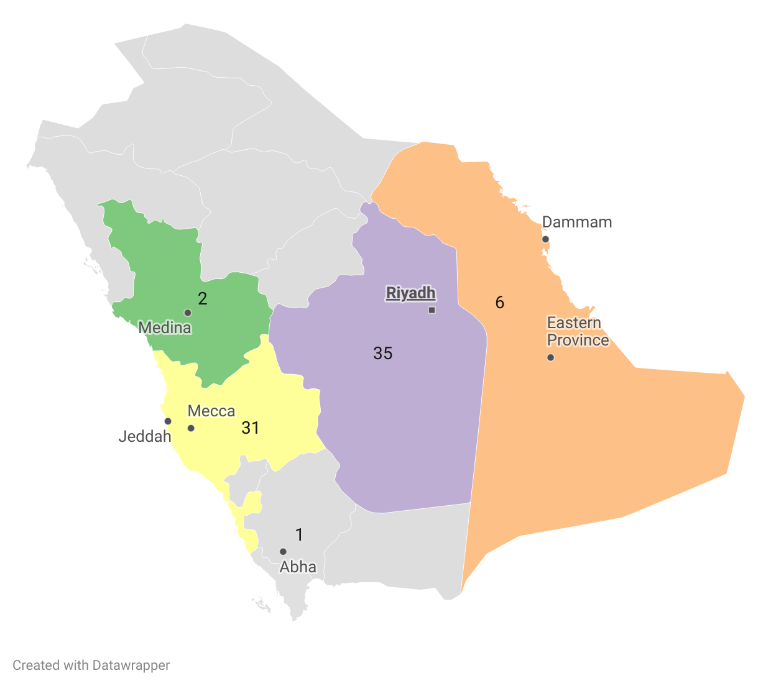


3B

**Figure 4: Percentage of clinical trial phases and BE studies at inspection visits**

**Figure 5: Therapeutic areas and phases of inspected facilities**

**Table 3: GCP Distribution of findings at clinical investigator site inspections**

| **GCP distribution of findings** | **Critical** | **Major** | **Other** | **Comment** | **Grand Total** |
| --- | --- | --- | --- | --- | --- |
| Investigator | 27 | 54 | 47 | 5 | 133 |
| Clinical trial protocol | 28 | 25 | 16 | 2 | 71 |
| Essential Documents | 15 | 17 | 13 | 3 | 48 |
| Regulatory requirement | 13 | 16 | 1 | 1 | 30 |
| Sponsor | 4 | 15 | 4 | 2 | 25 |
| The principles | 4 | 1 | - | - | 5 |
| Investigators Brochure | 1 | - | - | - | 1 |
| **Grand Total** | **91** | **128** | **81** | **13** | **313** |

**Figure 6: GCP observations and grades at clinical investigator sites**

**Table 4: GCP distribution of findings at CRO inspections**

| **GCP distribution of findings** | **Critical** | **Major** | **Other** | **Comment** | **Grand Total** |
| --- | --- | --- | --- | --- | --- |
| Regulatory requirement | 25 | 20 | 11 |  | 56 |
| Sponsor | 13 | 17 | 13 | 1 | 44 |
| Essential Documents | 5 | 2 | - | - | 7 |
| Investigator | - | 3 | 2 | 1 | 6 |
| The principles | 5 | - | - | - | 5 |
| **Grand Total** | **48** | **42** | **26** | **2** | **118** |

**Figure 7: GCP observations and grades at CROs**

**Table 5: GCP distribution of findings at BE inspections**

| **GCP distribution of finding** | **Critical** | **Major** | **Other** | **Comment** | **Grand Total** |
| --- | --- | --- | --- | --- | --- |
| Sponsor | 20 | 44 | 33 | 22 | 119 |
| Regulatory requirement | 15 | 30 | 18 | 5 | 68 |
| Investigator | 8 | 11 | 11 | 4 | 34 |
| The principles | 6 | 9 | 4 | - | 19 |
| Clinical trial protocol | 6 | 3 | - | 1 | 10 |
| Essential Documents | 2 | 2 | 1 | - | 5 |
| Investigators Brochure | - | 1 | - | - | 1 |
| **Grand Total** | **57** | **100** | **67** | **32** | **256** |

**Figure 8:GCP observations and grades at BEs**

**Table 6: GCP distribution of findings at phase I units inspections**

| **GCP distribution of finding** | **Critical** | **Major** | **Other** | **Comment** | **Grand Total** |
| --- | --- | --- | --- | --- | --- |
| Regulatory requirement | 2 | 10 | 2 | 1 | 15 |
| Sponsor | 3 | 6 | 1 | 1 | 11 |
| Investigator | 2 | 3 | 3 | - | 8 |
| Essential Documents | - | 1 | - | - | 1 |
| **Grand Total** | **7** | **20** | **6** | **2** | **35** |

**Figure 9: GCP observations and grades at phase 1 units**

1. International Council for Harmonisation Of Technical Requirements for Pharmaceuticals for Human Use (Ich) Ich Harmonised Guideline Integrated Addendum to ICH E6(R1): Guideline for Good Clinical Practice E6(R2). 2016. [↑](#footnote-ref-1)
2. Classification of GMP Deficiencies [Internet]. 2019. Available from: http://www.picscheme.org [↑](#footnote-ref-2)
